# Intra-Articular Lactate Dehydrogenase A Inhibitor Oxamate Reduces Experimental Osteoarthritis and Nociception in Rats via Possible Alteration of Glycolysis-Related Protein Expression in Cartilage Tissue

**DOI:** 10.3390/ijms241310770

**Published:** 2023-06-28

**Authors:** Zhi-Hong Wen, Chun-Sung Sung, Sung-Chun Lin, Zhi-Kang Yao, Yu-Cheng Lai, Yu-Wei Liu, Yu-Yan Wu, Hsi-Wen Sun, Hsin-Tzu Liu, Wu-Fu Chen, Yen-Hsuan Jean

**Affiliations:** 1 Department of Marine Biotechnology and Resources, National Sun Yat-Sen University, Kaohsiung 80424, Taiwan; wzh@mail.nsysu.edu.tw (Z.-H.W.); akang329@vghks.gov.tw (Z.-K.Y.); d52449@auh.org.tw (Y.-C.L.); o520184o@gmail.com (Y.-W.L.); yywu@gapp.nthu.edu.tw (Y.-Y.W.); suncwen@ntut.edu.tw (H.-W.S.); ma4949@cgmh.org.tw (W.-F.C.); 2 Institute of BioPharmaceutical Sciences, National Sun Yat-Sen University, Kaohsiung 80424, Taiwan; 3 Division of Pain Management, Department of Anesthesiology, Taipei Veterans General Hospital, Taipei 112201, Taiwan; sung6119@gmail.com; 4 School of Medicine, National Yang Ming Chiao Tung University, Taipei 112304, Taiwan; 5 Department of Orthopedic Surgery, Pingtung Christian Hospital, No. 60 Dalian Road, Pingtung 90059, Taiwan; 02572@ptch.org.tw; 6 Department of Orthopedic Surgery, Kaohsiung Veterans General Hospital, Kaohsiung 81341, Taiwan; 7 Department of Orthopedics, Asia University Hospital, Taichung 41354, Taiwan; 8 Department of Medical Research, Hualien Tzu Chi Hospital, Buddhist Tzu Chi Medical Foundation, Hualien 97002, Taiwan; hsintzu_liu@tzuchi.com.tw; 9 Department of Neurosurgery, Kaohsiung Chang Gung Memorial Hospital and Chang Gung University College of Medicine, Kaohsiung 833301, Taiwan

**Keywords:** osteoarthritis, oxamate, glycolysis, chondrocytes, lactate dehydrogenase A

## Abstract

Osteoarthritis (OA) is the most common form of arthritis and joint disorder worldwide. Metabolic reprogramming of osteoarthritic chondrocytes from oxidative phosphorylation to glycolysis results in the accumulation of lactate from glycolytic metabolite pyruvate by lactate dehydrogenase A (LDHA), leading to cartilage degeneration. In the present study, we investigated the protective effects of the intra-articular administration of oxamate (LDHA inhibitor) against OA development and glycolysis-related protein expression in experimental OA rats. The animals were randomly allocated into four groups: Sham, anterior cruciate ligament transection (ACLT), ACLT + oxamate (0.25 and 2.5 mg/kg). Oxamate-treated groups received an intra-articular injection of oxamate once a week for 5 weeks. Intra-articular oxamate significantly reduced the weight-bearing defects and knee width in ACLT rats. Histopathological analyses showed that oxamate caused significantly less cartilage degeneration in the ACLT rats. Oxamate exerts hypertrophic effects in articular cartilage chondrocytes by inhibiting glucose transporter 1, glucose transporter 3, hexokinase II, pyruvate kinase M2, pyruvate dehydrogenase kinases 1 and 2, pyruvate dehydrogenase kinase 2, and LHDA. Further analysis revealed that oxamate significantly reduced chondrocyte apoptosis in articular cartilage. Oxamate attenuates nociception, inflammation, cartilage degradation, and chondrocyte apoptosis and possibly attenuates glycolysis-related protein expression in ACLT-induced OA rats. The present findings will facilitate future research on LDHA inhibitors in prevention strategies for OA progression.

## 1. Introduction

Several complex mechanisms induce osteoarthritis (OA), such as the progressive erosion of articular cartilage, proteoglycan degradation, and disruption of the collagen network, leading to the gradual destruction of joints and loss of function [1]. OA can also be triggered by inflammation and has been associated with several inflammatory processes [2]. Previous studies have demonstrated that metabolic pathways undergo reprogramming in response to the changes in energy demand following an inflammatory response [3]. During inflammation, cells preferentially use glycolysis to generate energy; however, it has been shown that metabolic disorders of glycolysis could result in chondrocyte hypertrophy and extracellular matrix (ECM) degradation [4]. Furthermore, OA disrupts the typical energy metabolism pathways in chondrocytes; this disruption is characterized by enhanced glycolysis and decreased oxidative phosphorylation (OXPHOS) capacity [5,6,7].

Recent studies have reported correlations between glycolysis and various diseases, including neurodegenerative diseases [8], rheumatoid arthritis [9], and cancer [10]. It has been known that glycolysis-related proteins or enzymes, such as glucose transporter 1 (GLUT1) [11], pyruvate kinase M2 (PKM2) [10], and lactate dehydrogenase A (LHDA) [12], are upregulated in the chondrocytes of patients with OA compared to those from healthy controls. Interestingly, Qu et al. (2015) demonstrated that while glycolysis is upregulated in OA chondrocytes, ATP energy acquisition is decreased, thereby inhibiting chondrocyte proliferation, differentiation, and cell viability [13]. The glycolytic intermediate pyruvate is converted to lactate by LHDA. This conversion pathway generates only two ATP molecules, providing much less energy than OXPHOS; furthermore, the lactate accumulation results in an acidic extra-cellular microenvironment [14] in the OA articular cavity, which causes pain and cartilage degeneration [15,16,17,18]. Lactate, once thought to be an end product of glycolysis, has recently emerged as a critical regulator of OA development [19]. Inhibitory studies of LHDA activity in chondrocytes through genetic (LDHA knockout mice) or pharmacological manipulation in vitro have identified LDHA as a potential therapeutic target for OA treatment [12].

Oxamate is a pyruvate analog that acts as a competitive inhibitor of LHDA to block the conversion of pyruvate to lactate [20]. It inhibits glycolysis and stimulates mitochondrial OXPHOS to promote the differentiation of mouse osteoprogenitors and human bone marrow stromal cells [21]. However, to our knowledge, no studies have examined oxamate’s protective effects against OA. In the present study, we evaluated the therapeutic effects of LDHA inhibitor oxamate on OA development and chondrocyte apoptosis and elucidated the role of glycolysis-related protein expression of cartilage tissues in anterior cruciate ligament transection (ACLT)-induced OA rats.

## 2. Results

### 2.1. Effects of Intra-Articular Oxamate Administration on ACLT-Induced Nociception

**Figure 1:**
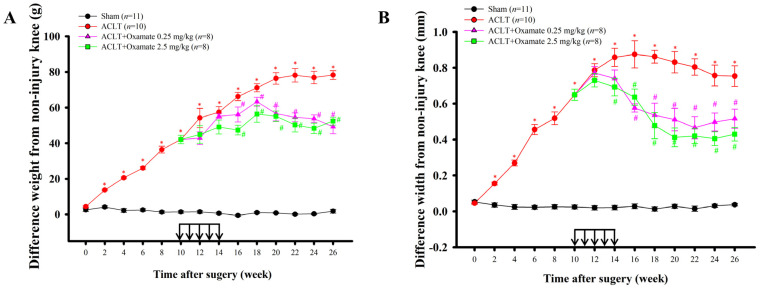
Effects of intra-articular oxamate injection on ACLT-induced OA. The effects of oxamate on (**A**) ACLT-induced hind limb weight-bearing deficits and (**B**) knee swelling were studied over time. Rats in the ACLT + oxamate groups were intra-articularly injected with oxamate (0.25 or 2.5 mg/kg per week, black arrow) from the 10th to 14th week after ACLT. Data are expressed as means ± SEM for each group. (* *p* < 0.05, compared with the sham group; # *p* < 0.05, compared with the ACLT group). ACLT: anterior cruciate ligament transection.

To assess the effects of oxamate on OA-induced weight-bearing changes and knee swelling (change in knee joint width), 0.25 and 2.5 mg/kg oxamate was administered via intra-articular injection once a week from week 10 to week 14 after ACLT or sham surgery. In addition to the pain and joint instability caused by uneven weight-bearing, rats with OA experience an inflammatory response that includes joint swelling. Neurological locomotor function was analyzed using the Basso, Beattie, and Bresnahan (BBB) rating scale to assess the motor effects of the sham, ACLT, ACLT + 0.25 mg/kg oxamate, and ACLT + 2.5 mg/kg oxamate groups. All the groups exhibited a normal neurological profile (BBB score = 25) and showed no obvious neurological defects after intra-articular oxamate injection. After ACLT, weight-bearing changes exhibited a gradual increase, reaching a peak prior to the initiation of oxamate treatment at week 10 (Figure 1A). The hind limbs of rats in the ACLT group (42.08 ± 2.40 g) showed significantly greater weight-bearing changes than those in the sham group (1.41 ± 0.75 g) (*p* < 0.001). A comparison of the ACLT + oxamate 0.25 mg/kg group and ACLT + oxamate 2.5 mg/kg group with the ACLT group demonstrated significant differences in the weight-bearing changes starting from after week 16 (*p* = 0.045; *p* < 0.001, respectively) and persisting until week 26. Based on the above results, 0.25 and 2.5 mg/kg oxamate can effectively improve lower-limb weight-bearing distribution in the ACLT-induced OA model. After ACLT, knee joint swelling increased in rats until the initiation of oxamate treatment at week 10 (Figure 1B). Rats in the ACLT group (0.65 ± 0.03 mm) exhibited significantly greater knee joint swelling than those in the sham group (0.02 ± 0.01 mm) (*p* < 0.001). The comparison of the ACLT + oxamate 0.25 mg/kg and ACLT + oxamate 2.5 mg/kg treatment group with the ACLT group demonstrated significant differences starting from after week 16 (*p* = 0.004; *p* = 0.035, respectively) and persisting until week 26. Based on the above results, 0.25 and 2.5 mg/kg oxamate effectively improved knee joint swelling in the ACLT-induced OA model.

### 2.2. Oxamate Attenuates Cartilage Degradation in ACLT-Rats

**Figure 2:**
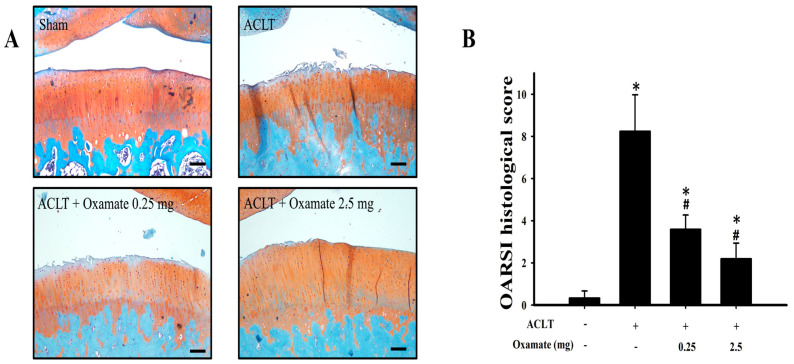
Histopathological evaluation of knee joints after oxamate treatment in ACLT-rats. (**A**) Histological sections of knee joints from the sham, ACLT, and ACLT + oxamate (0.25 or 2.5 mg/kg) groups were stained with Safranin O/Fast Green. (**B**) Histopathological quantitative changes in the knee joints of the four studied groups were evaluated using the OARSI scoring system. The histogram shows the OARSI scores of the sham, ACLT, and ACLT + oxamate (0.25 or 2.5 mg/kg) groups. Data are expressed as means ± SEM for each group. The scale bar represents 100 μm. (* *p* < 0.05, compared with the sham group; # *p* < 0.05, compared with the ACLT group). OARSI: Osteoarthritis Research Society International.

Safranin O/Fast Green staining was used to assess the protective effects of oxamate against cartilage degradation and damage, and the OARSI system was used to analyze the knee joint tissues in the experimental groups. The results are shown in Figure 2A. Compared to the sham group, greater ECM loss and severe damage to the articular surface were observed in the ACLT group. Although ECM loss was not alleviated in the ACLT + oxamate 0.25 mg/kg group, articular surface damage was improved. ECM loss and articular surface injury improved in the ACLT + oxamate 2.5 mg/kg group. Figure 2B shows the quantitative analysis results by the OARSI system: The ACLT group (8.25 ± 1.73) scored significantly higher than the sham group (0.33 ± 0.33) (*p* = 0.001). The OARSI score was considerably lower in the ACLT + oxamate 0.25 mg/kg group (3.60 ± 0.68) than in the ACLT group (*p* = 0.045), while the ACLT + oxamate 2.5 mg/kg group (2.20 ± 0.73) had a significantly lower OARSI score than the ACLT group (*p* = 0.015). Based on the above results, treatment with 0.25 and 2.5 mg/kg oxamate can significantly reduce cartilage ECM loss and articular surface injury in the ACLT-induced OA model.

### 2.3. Oxamate Affects Glucose Transporter 1- and 3 Expression in ACLT-Cartilage

**Figure 3:**
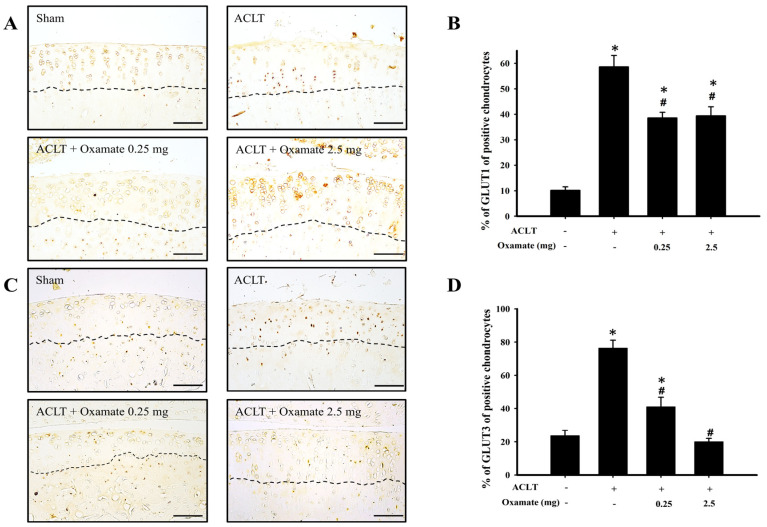
The effect of oxamate on glucose transporter 1 and glucose transporter 3 expression in cartilage tissues after ACLT. Immunohistochemical analysis of (**A**) glucose transporter 1 and (**B**) glucose transporter 3 in knee joint sections from the sham, ACLT, and ACLT + oxamate (0.25 or 2.5 mg/kg) groups. The quantitative analysis of the ratio of (**C**) glucose transporter 1-positive and (**D**) glucose transporter 3-positive cells in joint sections is presented. Data are expressed as means ± SEM for each group. The scale bar represents 100 μm. (* *p* < 0.05, compared with the sham group; # *p* < 0.05, compared with the ACLT group). GLUT: glucose transporter.

Glucose transporters 1 and 3 are isoforms. Their expression indicates an increased glucose uptake and, therefore, enhanced glycolysis. The results are shown in Figure 3A. The ACLT group exhibited increased glucose transporter 1 protein expression in the cartilage tissues compared to the sham group. Treatment with 0.25 and 2.5 mg/kg oxamate significantly decreased glucose transporter 1 protein expression in ACLT-cartilage tissues. The quantitative results are shown in Figure 3B. The expression of glucose transporter 1 protein was significantly higher in the ACLT group than in the sham group (*p* < 0.001). After treatment with 0.25 mg/kg (*p* = 0.007) and 2.5 mg/kg (*p* = 0.016) oxamate, glucose transporter 1 protein expression was significantly downregulated compared to that in the ACLT group. The above results showed that after treatment with 0.25 and 2.5 mg/kg oxamate, there was a decreased expression of glucose transporter 1 protein in ACLT-cartilage tissues. The results of glucose transporter 3 protein expression are shown in Figure 3C. Compared to the sham group, glucose transporter 3 protein expression was increased in the cartilage tissues in the ACLT group. Treatment with 0.25 and 2.5 mg/kg oxamate significantly decreased glucose transporter 3 protein expression in ACLT-cartilage tissues. Quantitative results are shown in Figure 3D. Glucose transporter 3 protein expression was significantly higher in the ACLT group than in the sham group (*p* < 0.001). After treatment with 0.25 (*p* = 0.003) and 2.5 mg/kg (*p* < 0.001) oxamate, the expression of glucose transporter 3 protein was significantly downregulated compared to the ACLT group. The above results show that at both concentrations, oxamate can decrease glucose transporter 3 protein expression in ACLT-cartilage tissues.

### 2.4. Oxamate Affects Hexokinase-II Expression in ACLT-Cartilage

**Figure 4:**
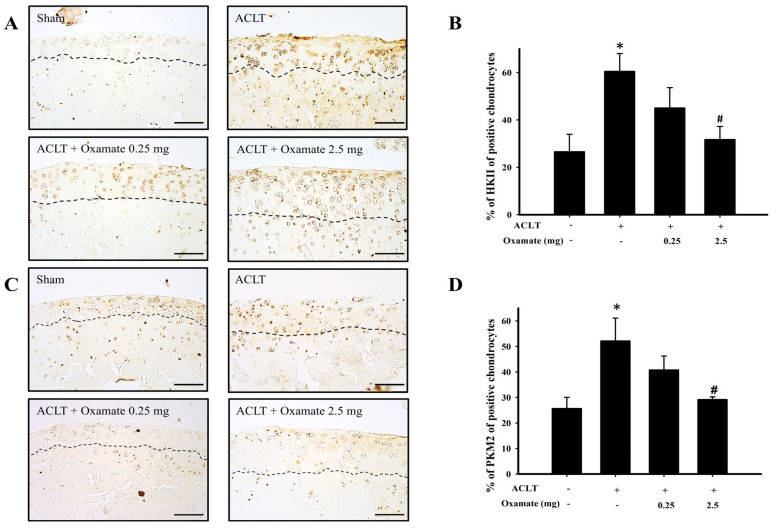
Effect of oxamate on hexokinase-II and pyruvate kinase M2 expression in cartilage tissues after ACLT. Immunohistochemical analysis of (**A**) hexokinase-II and (**C**) pyruvate kinase M2 in joint sections from the sham, ACLT, and ACLT + oxamate (0.25 or 2.5 mg/kg) groups. The quantitative analysis of the ratio of (**B**) hexokinase II-positive and (**D**) pyruvate kinase M2-positive cells in joint sections is presented. Data are expressed as means ± SEM for each group. The scale bar represents 100 μm. (* *p* < 0.05, compared with the sham group; # *p* < 0.05, compared with the ACLT group). HK: hexokinase; PK: pyruvate kinase.

Hexokinase-II is a major regulatory protein in glycolysis, with its expression increasing the conversion rate of glucose to glucose 6-phosphate and thereby promoting glycolysis (Figure 4A). Compared with the sham group, hexokinase-II protein expression was increased in the cartilage tissues in the ACLT group. Treatment with 2.5 mg/kg oxamate decreased hexokinase-II protein expression in ACLT-cartilage (Figure 4B). Compared with the sham group, hexokinase-II protein expression was significantly increased in the ACLT group (*p* = 0.032). Although hexokinase-II protein expression was downregulated after treatment with 0.25 mg/kg oxamate compared to that in the ACLT group (*p* = 0.246), the decrease was not significant. However, hexokinase-II protein expression was significantly downregulated after treatment with 2.5 mg/kg oxamate compared with the ACLT group (*p* = 0.036). Based on the above results, treatment with 2.5 mg/kg oxamate significantly decreased hexokinase-II protein expression in the cartilage tissues of ACLT-induced OA rats.

### 2.5. Oxamate Affects Pyruvate Kinase M2 Expression in ACLT Cartilage

Pyruvate kinase M2, a pyruvate kinase isoform, plays a multifaceted role in energy metabolism by aiding cells in harnessing energy in hypoxic environments via glycolysis (Figure 4C). Compared with the sham group, pyruvate kinase M2 protein expression increased in cartilage tissues (above the dotted line) in the ACLT group. Oxamate 2.5 mg/kg decreased pyruvate kinase M2 protein expression in ACLT-cartilage (Figure 4D). Compared with the sham group, pyruvate kinase M2 protein expression was significantly increased in the ACLT group (*p* = 0.037). Although pyruvate kinase M2 protein expression was downregulated after treatment with oxamate 0.25 mg/kg compared to that in the ACLT group (*p* = 0.318), the decrease was not significant. However, in the present study, pyruvate kinase M2 protein expression was significantly downregulated after treatment with oxamate 2.5 mg/kg compared to that in the ACLT group (*p* = 0.043). Based on the above results, treatment with 2.5 mg/kg oxamate significantly decreased pyruvate kinase M2 protein expression in the cartilage tissues of ACLT-induced OA rats.

### 2.6. Oxamate Affects Pyruvate Dehydrogenase Kinase 1- and 2 Expression in ACLT-Cartilage

**Figure 5:**
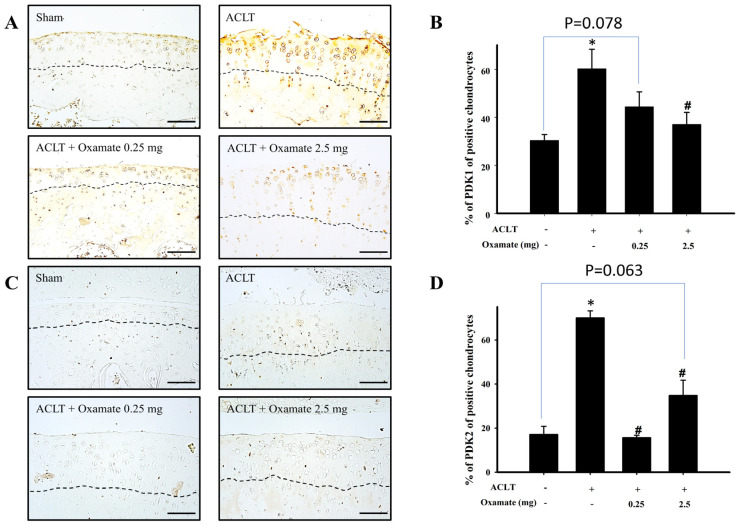
Effect of oxamate on pyruvate dehydrogenase kinase 1 and pyruvate dehydrogenase kinase 2 expression in cartilage tissues after ACLT administration. Immunohistochemical analysis of (**A**) pyruvate dehydrogenase kinase 1 and (**C**) pyruvate dehydrogenase kinase 2 in joint sections from the sham, ACLT, and ACLT + oxamate (0.25 or 2.5 mg/kg) groups. The quantitative analysis of the ratio of (**B**) pyruvate dehydrogenase kinase 1-positive and (**D**) pyruvate dehydrogenase kinase 2-positive cells in joint sections is presented. Data are expressed as means ± SEM for each group. The scale bar represents 100 μm. (* *p* < 0.05, compared with the sham group; # *p* < 0.05, compared with the ACLT group). PDK: pyruvate dehydrogenase kinase.

The pyruvate dehydrogenase kinases 1 and 2 isoforms primarily function as an inhibitor of acetyl-CoA entry into the tricarboxylic acid cycle; this inhibition results in the decrease of oxidative phosphorylation (Figure 5A). Compared with the sham group, pyruvate dehydrogenase kinase 1 protein expression in the ACLT group was increased in cartilage tissues. Treatment with 2.5 mg/kg oxamate decreased pyruvate dehydrogenase kinase 1 protein expression in ACLT-cartilage tissues (Figure 5B). Compared with the sham group, pyruvate dehydrogenase kinase 1 protein expression was significantly increased in the ACLT group (*p* = 0.012). Although pyruvate dehydrogenase kinase 1 protein expression was downregulated after treatment with 0.25 mg/kg oxamate compared to that in the ACLT group (*p* = 0.171), the decrease was not significant. However, pyruvate dehydrogenase kinase 1 protein expression was significantly downregulated after treatment with oxamate 2.5 mg/kg compared to the ACLT group (*p* = 0.050). Based on the results, treatment with 2.5 mg/kg oxamate significantly decreased pyruvate dehydrogenase kinase 1 protein expression in the ACLT-induced OA model cartilage tissues. The pyruvate dehydrogenase kinase 2 protein expression results are shown in Figure 5C. Compared to the sham group, pyruvate dehydrogenase kinase 2 protein expression was increased in cartilage tissues in the ACLT group. Treatment with 0.25 and 2.5 mg/kg oxamate decreased pyruvate dehydrogenase kinase 2 protein expression in the ACLT-cartilage tissues (Figure 5D). Compared with the sham group, pyruvate dehydrogenase kinase 2 protein expression was significantly increased in the ACLT group (*p* < 0.001). After treatment with 0.25 (*p* < 0.001) and 2.5 mg/kg (*p* = 0.004) oxamate, pyruvate dehydrogenase kinase 2 protein expression was significantly downregulated compared to that in the ACLT group. The above results showed that oxamate at 0.25 and 2.5 mg/kg could decrease pyruvate dehydrogenase kinase 2 protein expression in ACLT-cartilage tissues.

### 2.7. Oxamate Affects Lactate Dehydrogenase a Expression in ACLT-Cartilage

**Figure 6:**
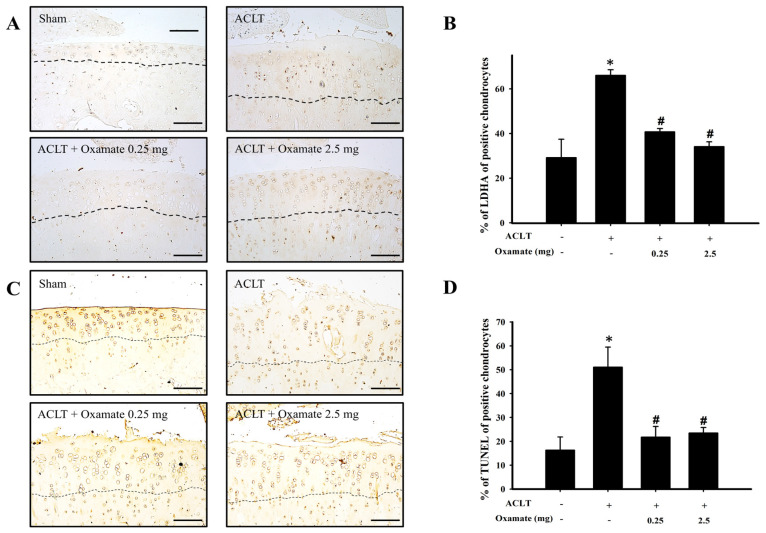
Effect of oxamate LDHA expression and TUNEL staining in cartilage tissues after ACLT administration. (**A**) Immunohistochemical analysis of LDHA and (**C**) TUNEL staining in joint sections from the sham, ACLT, and ACLT + oxamate (0.25 or 2.5 mg/kg) groups. The quantitative analysis of the ratio of (**B**) LDHA-positive and (**D**) TUNEL-positive cells in joint sections is presented. Data are expressed as means ± SEM for each group. The scale bar represents 100 μm. (* *p* < 0.05, compared with the sham group; # *p* < 0.05, compared with the ACLT group). LDHA: lactate dehydrogenase A; TUNEL: terminal deoxynucleotidyl transferase dUTP nick-end labeling.

Lactate dehydrogenase A catalyzes the conversion of pyruvate to lactate during the anaerobic glycolytic pathway (Figure 6A). The ACLT group exhibited increased lactate dehydrogenase A protein expression in cartilage tissues compared to the sham group. Treatment with 0.25 and 2.5 mg/kg oxamate decreased lactate dehydrogenase A protein expression in ACLT-cartilage tissues (Figure 6B). Lactate dehydrogenase A protein expression was significantly higher in the ACLT group than in the sham group (*p* = 0.013). After treatment with 0.25 (*p* < 0.001) and 2.5 mg/kg (*p* < 0.001) oxamate, lactate dehydrogenase A protein expression was significantly downregulated compared to that in the ACLT group. The above results showed that 0.25 and 2.5 mg/kg oxamate could decrease lactate dehydrogenase A protein expression in ACLT-cartilage tissues.

### 2.8. Oxamate Affects TUNEL Expression (Apoptosis) in ACLT-Cartilage

The ACLT group exhibited increased terminal deoxynucleotidyl transferase dUTP nick end labeling (TUNEL) expression in the cartilage tissues compared to the sham group. The results are shown in Figure 6C. Treatment with 0.25 and 2.5 mg/kg oxamate significantly decreased TUNEL protein expression in ACLT-cartilage tissues (Figure 6D). TUNEL staining was significantly higher in the ACLT group than in the sham group (*p* = 0.013). After treatment with 0.25 (*p* = 0.022) and 2.5 mg/kg (*p* = 0.019) oxamate, TUNEL protein expression was significantly downregulated compared to that in the ACLT group. The above results demonstrated that 0.25 and 2.5 mg/kg oxamate can decrease TUNEL protein expression in ACLT-cartilage tissues.

## 3. Discussion

In the present study, the intra-articular administration of oxamate to rats with ACLT-induced OA significantly improved weight-bearing defects, knee joint swelling, and synovitis in the hind limbs; oxamate attenuated ACLT-induced cartilage degradation and chondrocyte apoptosis improvements were also seen. Concurrently, oxamate decreased the upregulation of glycolysis-related proteins, such as glucose transporter 1- and 3, hexokinase-II, pyruvate kinase M2, pyruvate dehydrogenase kinase 1- and 2, and LDHA, in the cartilage tissues of OA rats. Our study is the first to demonstrate in vivo the chondroprotective role of oxamate in an experimentally induced OA rat model.

According to a clinical study, there is a 50% chance of developing knee OA 10–20 years after ACL injury [22]. Furthermore, several studies have demonstrated that OA progression is accompanied by nociceptive behaviors [23,24]. The ACLT-induced OA model allows for the clear observation of mechanical allodynia, changes in the weight-bearing distribution, and knee swelling in injured knees after ACLT [23,25]. In the present study, intra-articular injection of oxamate after ACLT attenuated the nociceptive behavior of weight-bearing defects in the hind limb, swelling of the knee joint, and synovitis (Figure 1). These results suggest that oxamate ameliorates nociception and inflammation in the OA model. In our preliminary observations, compared with the ACLT group, changes in the hind paw weight-bearing distribution decreased significantly, but knee joint swelling decreased insignificantly in the ACLT + oxamate 0.125 mg/kg group (*n* = 3). Therefore, in the present study, we examined the effects of 0.25 and 2.5 mg/kg oxamate on ACLT rats.

In previous inhibitory studies on cancer cell growth, oxamate has been shown to exert its action primarily through the regulation of glycolysis [26]. Oxamate promotes bone formation and strength in mice [21]; however, its protective mechanisms against OA are unknown. There is evidence that synovial membrane inflammation plays a significant role in OA [27]. The manifestations of inflammation associated with the ACLT model include joint effusion and synovial membrane hyperplasia [28], both of which are important factors in the development of joint pain in patients with OA [29]. In the present study, moderate synovitis and OA development were noted in the ACLT group, and treatment with either 0.25 or 2.5 mg/kg oxamate reduced the severity of OA (Figure 2). That oxamate can reduce synovitis and OA in an experimental rat model of OA is a novel finding of this study.

An oxygen-gradient microenvironment exists in cartilage tissues, and chondrocytes in the deepest part can experience oxygen concentrations as low as 1%. Normal chondrocytes meet 75% of their energy demands via glycolysis, with their mitochondria generating the remaining 25% via oxidative phosphorylation [30]. Hence, both glycolysis and oxidative phosphorylation occur in chondrocytes [31]. Although oxidative phosphorylation provides only 25% of the ATP requirement in chondrocytes, this pathway can synthesize 36 ATP molecules from a single glucose molecule, and its energy generation efficiency is far greater than that of glycolysis, which can only synthesize two ATP molecules from one glucose molecule [32]. OA causes changes in energy metabolism pathways in chondrocytes, which are characterized by enhanced glycolysis and decreased oxidative phosphorylation capacity [7]. Currently, 14 glucose transporter isoforms have been identified [33]. Their function is to transport extracellular glucose into the cells, thereby providing glucose for glycolysis. Studies have shown that glucose transporters 1-, 3, and 9 are expressed in normal chondrocytes, with glucose transporter 1 and 3 being extremely sensitive to hypoxic environments [34]. Increased transportation of glucose helps cells obtain energy; however, it also upregulates oxidative stress and inflammation [34], which can damage chondrocytes and ultimately exacerbate OA [31]. Vázquez-Mosquera et al. (2013) showed that the expression of glucose transporter 1 and 3 in the cartilage tissues of patients with OA was higher than in healthy individuals, but these differences were insignificant [11]. The present study showed that glucose transporter 1 and 3 expression in cartilage tissue was significantly upregulated in the ACLT-induced OA group compared to that in the sham group (Figure 3). Intra-articular injection of oxamate inhibited the ACLT-induced expression of glucose transporters 1 and 3 in chondrocytes (Figure 3). We hypothesized that oxamate downregulates the expression of these two glucose transporters; thus, oxamate may regulate energy generation more efficiently and reduce oxidative stress and inflammation, both of which have protective effects against OA.

The primary function of hexokinase-II is to catalyze the conversion of phosphorylated glucose to glucose 6-phosphate [35]. Additionally, hexokinase-II has been shown to reprogram tumor cell metabolism to aerobic glycolysis [36]. A previous study revealed that hexokinase-II expression in peripheral blood mononuclear cells is higher in patients with OA than in healthy individuals [37]. TGF-β1 upregulates hexokinase-II protein expression in OA chondrocytes, induces aerobic glycolysis, increases glucose consumption and lactate synthesis, and decreases ATP synthesis simultaneously [38]. The present study found that ACLT upregulated hexokinase-II expression in chondrocytes (Figure 4A,B). Intra-articular injection of oxamate inhibited ACLT-induced hexokinase-II upregulation in chondrocytes. Therefore, our findings suggest that ACLT-induced upregulation of hexokinase -II may be regulated by oxamate.

Pyruvate kinase has two isoforms, pyruvate kinase M1 and 2, and mainly mediates one of the terminal reactions in glycolysis: the conversion of phosphoenolpyruvate to pyruvate, which generates one ATP [10]. Previous studies have proven that pyruvate kinase 2 expression in the cartilage of patients with OA is higher than that of healthy individuals and that pyruvate kinase 2 knockdown may downregulate glucose transporter 1, hypoxia-induced factor-1α, and LDHA expression, inhibit OA chondrocyte proliferation, and promote apoptosis [39]. The present study found that pyruvate kinase M2 was significantly increased in the chondrocytes of ACLT rats and that oxamate intra-articular injection significantly downregulated the expression of this protein (Figure 4C,D). This could be because oxamate may play a role in the oxidative phosphorylation pathway while partially restoring ATP synthesis. The pyruvate dehydrogenase complex is essential for aerobic metabolism because it catalyzes the entry of pyruvate into the mitochondria for oxidative phosphorylation [40]. The activity of the pyruvate dehydrogenase complex is negatively regulated by pyruvate dehydrogenase kinases. Under pathological conditions (e.g., diabetes, cancer, and sepsis), pyruvate dehydrogenase kinase activation inhibits pyruvate dehydrogenase complex activity and prevents cytoplasmic pyruvate from entering the mitochondria for oxidative phosphorylation; thus, decreasing ATP synthesis [41]. A hypoxic environment can increase the expression of hypoxia-inducible factor 1-α and activate pyruvate dehydrogenase kinase 1 [42]. Yudoh et al. (2004) demonstrated that hypoxia-induced factor-1α expression is elevated in the cartilage tissues of patients with OA and contributes to cartilage degeneration [43]. This results in the reprogramming of glucose metabolism from oxidative phosphorylation to anaerobic glycolysis, which decreases ATP synthesis [44]. The present study found that ACLT upregulated pyruvate dehydrogenase kinases 1- and 2 in chondrocytes (Figure 5), which may inhibit pyruvate dehydrogenase complex activity, thereby attenuating pyruvate entry into the mitochondrial oxidative phosphorylation for ATP synthesis. Treatment with 2.5 mg/kg oxamate decreased pyruvate dehydrogenase kinase 1 and 2 protein expression in ACLT-cartilage tissues. Therefore, we believe that oxamate may partially restore ATP synthesis via the oxidative phosphorylation pathway.

During the anaerobic phase, chondrocytes in an OA environment synthesize pyruvate via glycolysis, and LDHA converts pyruvate into lactate. This conversion pathway generates two ATP molecules; however, lactate accumulation creates an acidic microenvironment [14]. Extracellular pH < 7.1 inhibits ECM synthesis while increasing the expression of ECM-degrading enzymes, resulting in cartilage degeneration [16]. Arra et al. (2020) used an ex vivo OA model to demonstrate that inflammation induces chondrocyte glycolysis, elevates LDHA levels, and decreases the rate of oxidative phosphorylation [12]. Downregulation of LDHA can reduce the expression of matrix metalloproteinase-13 and has been shown to protect cartilage tissues in animal experiments [12]. The present study showed that in ACLT-induced OA rats, LDHA protein expression was upregulated in chondrocytes. Following oxamate injection, ECM loss and cartilage surface injury were significantly improved, and oxamate significantly inhibited LDHA protein expression in ACLT-induced OA (Figure 6A,B).

Chondrocyte apoptosis plays a key role in the degeneration and degradation of articular cartilage in OA [45]. Aberrant apoptosis, ECM integrity, and inflammatory responses in chondrocytes are related to cartilage degradation in OA [46]. Depending on the stimulus, apoptosis can occur via two main pathways: the death receptor-mediated pathway (extrinsic) and the mitochondrial-mediated pathway (intrinsic) [47]. Moreover, the number of apoptotic chondrocytes significantly increased in OA, which is closely related to the endoplasmic reticulum and death receptor pathways [48]. In the present study, the ACLT + oxamate-treated group showed a significantly reduced percentage of TUNEL-positive chondrocytes compared to the ACLT group (Figure 6C,D). That oxamate can reduce chondrocyte apoptosis in an experimental rat model of OA is a novel finding of this study.

This study has some limitations. The first limitation is that surgically induced OA animal models may not entirely represent spontaneous, naturally occurring human OA [49]. In the present study, the surgically induced OA model by ACLT is representative of post-traumatic OA [22]. However, it has been well-documented that several factors contribute to OA development, including inflammation, trauma, aging, obesity, chondrocyte differentiation, and genetic predisposition [50]. Determining which OA animal models accurately represent patients with OA is crucial. In the future, the successful translation of therapies from the laboratory to clinical applications depends on the use of appropriate preclinical disease models. Thus, it is necessary to conduct experiments in different animal models. The second limitation is the administrative route of oxamate. Although intra-articular injection has the advantage of directly targeting the OA joint, there are technical aspects (such as injection volume and osmotic pressure) and risks (such as infection) that must be considered in its future clinical use. In further studies, we will attempt to devise an oral administration method to examine the protective effects of oxamate on OA. The third limitation involves the question of whether intra-articular oxamate alleviated subchondral bone structure remodeling after ACLT-induced OA. Although subchondral bone structure remodeling was not a primary objective of our present study, a previous study indicated that the subchondral bone structure in joint regions is a characteristic feature of OA histopathology and participates in load-bearing pressures on the body after cartilage degeneration [51]. Our previous study found that the bone surface and the trabecular number of subchondral bone were significantly higher and lower in the ACLT-knee joint, respectively [52]. The advantages of high-resolution and three-dimensional (3D) reconstruction in micro-computed tomography (CT) have been widely applied in the evaluation of animal and human OA histopathology of subchondral bone areas [53]. Therefore, another potential research direction is to utilize micro-CT to investigate the effect of oxamate on the changes of microarchitecture in subchondral bone after ACLT-induced OA of the knee.

## 4. Materials and Methods

### 4.1. Animals and Surgical Technique for OA Induction

OA was induced in male Wistar rats (8 weeks old, body weight 260–280 g) by ACLT of the right knee; surgery was not performed on the left knee. This procedure was modified from a previously described protocol [28,54]. Each animal was administered a subcutaneous pre-operative injection of enrofloxacin (5 mg/kg; Elanco Animal Health Korea Co., Ltd., Seoul, Republic of Korea) for prophylaxis. The animals were not immobilized after surgery and were allowed unrestricted cage activity. All rats were maintained in climate-controlled conditions on a 12-h light-dark cycle at 22–24 °C at a relative humidity of 50–55%.

### 4.2. Experimental Design and Oxamate Treatment

The rats were randomly allocated into the following four experimental groups: Group I: sham (*n* = 11); rats received arthrotomy without ACLT and were administered 50 μL of physiologically normal saline by intra-articular administration. Group II: ACLT (*n* = 10); animals underwent ACLT and were administered 50 μL of physiologically normal saline by intra-articular administration. Group III: ACLT + 0.25 mg/kg oxamate (*n* = 8); animals underwent ACLT and were administered 0.25 mg/kg of oxamate (Thermo Fisher Scientific, Waltham, MA, USA) by intra-articular administration once weekly for five consecutive weeks, beginning 10 weeks after surgery. Group IV: ACLT + 2.5 mg/kg oxamate (*n* = 8), animals underwent ACLT and were administered 0.25 mg/kg of oxamate, scheduled the same as group III. On the 26th week after surgery, the neurological locomotor function was analyzed using the BBB rating scale [55] to assess the motor effects of the sham, ACLT, ACLT + 0.25 mg/kg oxamate, and ACLT + 2.5 mg/kg oxamate groups. After placing rats into transparent Plexiglass boxes, two observers scored hind limb movements and walking behaviors for 4 min. Then, all animals were sacrificed, and their knee joints were collected for histopathological, immunohistochemical, and TUNEL analyses.

### 4.3. Test for Defects in Hind Limb Weight-Bearing and Knee Swelling (Change of Joint Width) Measurement

The effect of joint damage on the weight distribution in OA and contra-lateral knees was measured using an incapacitance meter tester (Singa Technology Corporation, Taipei, Taiwan) that independently measured the weight bearing of each hind paw. Changes in the distribution of hind paw weight-bearing were used as an index of joint discomfort as described previously [56,57]. The rats were placed in an angled Plexiglass chamber positioned so that each hind paw rested on a separate force plate. The force exerted by each hind limb (measured in grams) was averaged over a 5-s period. Each data point was the mean of three 5 s readings. The hind paw weight distribution was expressed as the differences in weight between the contralateral and ipsilateral limbs. The width of the knee joint was measured from the medial to the lateral aspects of the knee joint at approximately the level of the medial and lateral joint lines using a vernier caliper (AA847R, Aesculap, AG&CO, KG, Osnabrück, Germany). Changes in knee joint width, a measure of knee joint inflammation [58], were recorded weekly before and after ACLT for up to 26 weeks.

### 4.4. Gross Morphology and Histopathological Examination

At week 26 after ACLT, the rats were sacrificed by deep anesthesia with sodium pentobarbital (50 mg/kg; Health-Tech Parmaceutical Co., Ltd., Taipei, Taiwan), then perfused intracardially with heparin saline (0.1% heparin in 0.9% saline; 200 mL/rat) followed by freshly prepared 4% paraformaldehyde in 0.1 mol/L phosphate-buffered saline, pH 7.4. The joints were sectioned 1 cm above and below the joint line, fixed in 10% neutral buffered formalin for 3 days, and then decalcified for 8 weeks in a buffered 4% ethylenediaminetetraacetic acid and formalin solution. The joints were then sectioned mid-sagittally, washed under running tap water, and paraffin-embedded using an automatic processor (Autotechnicon Mono 2; Technion Co., Chauncey, NY, USA). Serial articular cartilage sections (1 μm) were cut on a rotatory microtome Microm HM340E (Walldorf, Germany) from the central weight-bearing surface of the femoral condyles and tibial plateau of both knees. Safranin-O/Fast Green staining was performed to assess the general morphology and matrix proteoglycan. Immediately after sacrifice, each knee was examined for gross morphologic changes in the cartilage lesions as described previously [59]. The articular cartilage was graded by microscopy according to the Osteoarthritis Research Society International (OARSI) system [60]. This system comprises six histological grades and four histological stages. The total score (score = grade × stage) ranges from 1 point (normal articular cartilage) to 24 points (no repair).

### 4.5. Immunohistochemistry and TUNEL Assay

Cartilage specimens were processed for immunohistochemical analysis as described previously [23,57]. Briefly, 1 μm sections of paraffin-embedded specimens were placed on slides, deparaffinized with xylene, and dehydrated in an alcohol gradient. The antigen was retrieved by enzymatic digestion with proteinase K (20 mM) in Tris-EDTA buffer for 45 min. The endogenous peroxidase activity was then quenched by 8 min incubation in 3% hydrogen peroxide. After washing three times for 8 min in Tween-tris-buffered saline (TTBS), the sections were incubated in phosphate-buffered saline (PBS) containing 4% normal horse serum for 60 min as a blocking agent for non-specific binding. The sections were incubated for 90 min with biotinylated anti-rabbit or -mouse IgG (Vector Labs, Burlingame, CA, USA) diluted 200-fold in 2% bovine serum albumin in PBS. The sections were then treated using the avidin-biotin complex technique using an ABC kit (Vectastain A.B.C. kit; Vector Labs). Primary antibodies used in the immunohistochemical analyses in the present study are shown in Table 1. The images were viewed using a Leica DM6000 microscope (Leica, Heidelberg, Germany) and captured using a FLEXACAM C1 microscope camera (Leica). The different antigens present in each cartilage specimen were quantified and estimated by determining the number of positively stained chondrocytes in the entire thickness of the cartilage, as described previously [61]. Apoptotic chondrocytes were detected by terminal deoxynucleotidyl transferase-mediated deoxyuridine triphosphate (d-UTP) nick end-labeling (TUNEL) assays. The samples were performed by using In Situ Cell Death Detection Kits, AP (Roche, Indianapolis, IN, USA) according to the manufacturer’s protocol. The cartilage was divided into six microscopic fields (three each in the superficial and deep zones) (magnification, 400×), and the results were averaged. For each OA specimen, before evaluation, the presence of an intact cartilage surface that could be detected and used as a marker for the morphometric analyses was identified. The data were expressed as the percentage of chondrocytes showing positive staining for the antigen (cell score), with the maximum cartilage specimen scores of 100%. Each slide was reviewed by two independent investigators blinded to the treatment groups. The data obtained from the medial and lateral femoral condyle and tibial plateau were considered together for the statistical analyses.

### 4.6. Data and Statistical Analysis

All continuous data are presented as means ± standard error of the mean (SEM). One-way analysis of variance (ANOVA) was used to test differences among the means of various scores in the experimental groups and for scores with significant differences. To compare mean differences between treatment and sham groups, Student–Newman–Keuls post hoc tests were used. The trends in the changes in nociceptive behavior and knee joint width were tested using repeated-measures ANOVA. Differences with *p* < 0.05 were considered significant, including the four groups in the present study.

## 5. Conclusions

In conclusion, oxamate may play an important role in the development of OA via possible modulation of glycolysis-related protein expression. Further studies are required to elucidate the mechanisms of action of oxamate in OA. An improved understanding of glycolytic metabolism during OA pathogenesis may lead to new insights into the etiology of OA and the development of metabolic-based therapeutic targets.

## Figures and Tables

**Table 1:** Primary antibodies used in the immunohistochemical analyses in the present study.

Primary Antibody	Host	Supplier	Catalog #	Dilution Ratio
GLUT1	Rabbit	Abcam, Cambridge, UK	ab652	1:1000
GLUT3	Rabbit	Biorbyt, Cambridge, UK	orb10727	1:1000
HK II	Rabbit	GeneTex, Irvine, CA, USA	gtx111525	1:3000
PKM2	Rabbit	Cell Signaling Technology, Danvers, MA, USA	4053s	1:800
PDK1	Mouse	Abcam	ab110025	1:1000
PDK2	Rabbit	Abcam	ab68164	1:800
LDHA	Rabbit	Novus, Littleton, CO, USA	NBP2-67483	1:500

## Data Availability

The datasets generated during and/or analyzed during the current study are available from the corresponding author upon reasonable request.
